# Small intra-individual variability of the pre-ejection period justifies the use of pulse transit time as approximation of the vascular transit

**DOI:** 10.1371/journal.pone.0204105

**Published:** 2018-10-10

**Authors:** Minke C. Kortekaas, Marit H. N. van Velzen, Frank Grüne, Sjoerd P. Niehof, Robert J. Stolker, Frank J. P. M. Huygen

**Affiliations:** Department of Anesthesiology, Erasmus University Medical Centre, Rotterdam, the Netherlands; Medical University Innsbruck, AUSTRIA

## Abstract

**Background:**

Vascular transit time (VTT) is the propagation time of a pulse wave through an artery; it is a measure for arterial stiffness. Because reliable non-invasive VTT measurements are difficult, as an alternative we measure pulse transit time (PTT). PTT is defined as the time between the R-wave on electrocardiogram and arrival of the resulting pulse wave in a distal location measured with photoplethysmography (PPG). The time between electrical activation of the ventricles and the resulting pulse wave after opening of the aortic valve is called the pre-ejection period (PEP), a component of PTT. The aim of this study was to estimate the variability of PEP at rest, to establish how accurate PTT is as approximation of VTT.

**Methods:**

PTT was measured and PEP was assessed with echocardiography (gold standard) in three groups of 20 volunteers: 1) a control group without cardiovascular disease aged <50 years and 2) aged >50 years, and 3) a group with cardiovascular risk factors, defined as arterial hypertension, dyslipidemia, kidney failure and diabetes mellitus.

**Results:**

Per group, the mean PEP was: 1) 58.5 ± 13.0 ms, 2) 52.4 ± 11.9 ms, and 3) 57.6 ± 11.6 ms. However, per individual the standard deviation was much smaller, i.e. 1) 2.0–5.9 ms, 2) 2.8–5.1 ms, and 3) 1.6–12.0 ms, respectively. There was no significant difference in the mean PEP of the 3 groups (p = 0.236).

**Conclusion:**

In conclusion, the intra-individual variability of PEP is small. A change in PTT in a person at rest is most probably the result of a change in VTT rather than of PEP. Thus, PTT at rest is an easy, non-invasive and accurate approximation of VTT for monitoring arterial stiffness.

## Introduction

Pulse transit time (PTT) is the sum of the pre-ejection period (PEP) and the vascular transit time (VTT). After opening of the aortic valve, a pulse pressure wave propagates with a certain speed through the blood vessels from the heart to a distal location; this is called the VTT. If an artery is stiff or has a small diameter (or both), the pulse wave will propagate faster and, subsequently, the VTT decreases. Vice versa, if the diameter of the artery increases, the pulse wave propagation is slower and the VTT increases. Therefore, the VTT is a measure for arterial stiffness. Increased stiffness can be either structural (age and atherosclerosis) or functional due to higher sympathetic activity or elevated blood pressure [1–3] It is a promising application for e.g. non-invasive continuous and cuffless blood pressure monitoring, which can also be used in children or in ambulatory setting [4–8].

Because reliable non-invasive VTT measurements are difficult, as an alternative we measure the PTT. PTT can be used to assess a successful loco regional block of an extremity [9, 10], to monitor vasomotor tone, and for assessment of autonomic nervous system response [11]. Another non-invasive measurement to estimate the VTT is the pulse wave velocity (PWV). However, determination of the arterial path length is necessary to calculate the PWV and this may create errors in the measurement [12].

**Fig 1:**
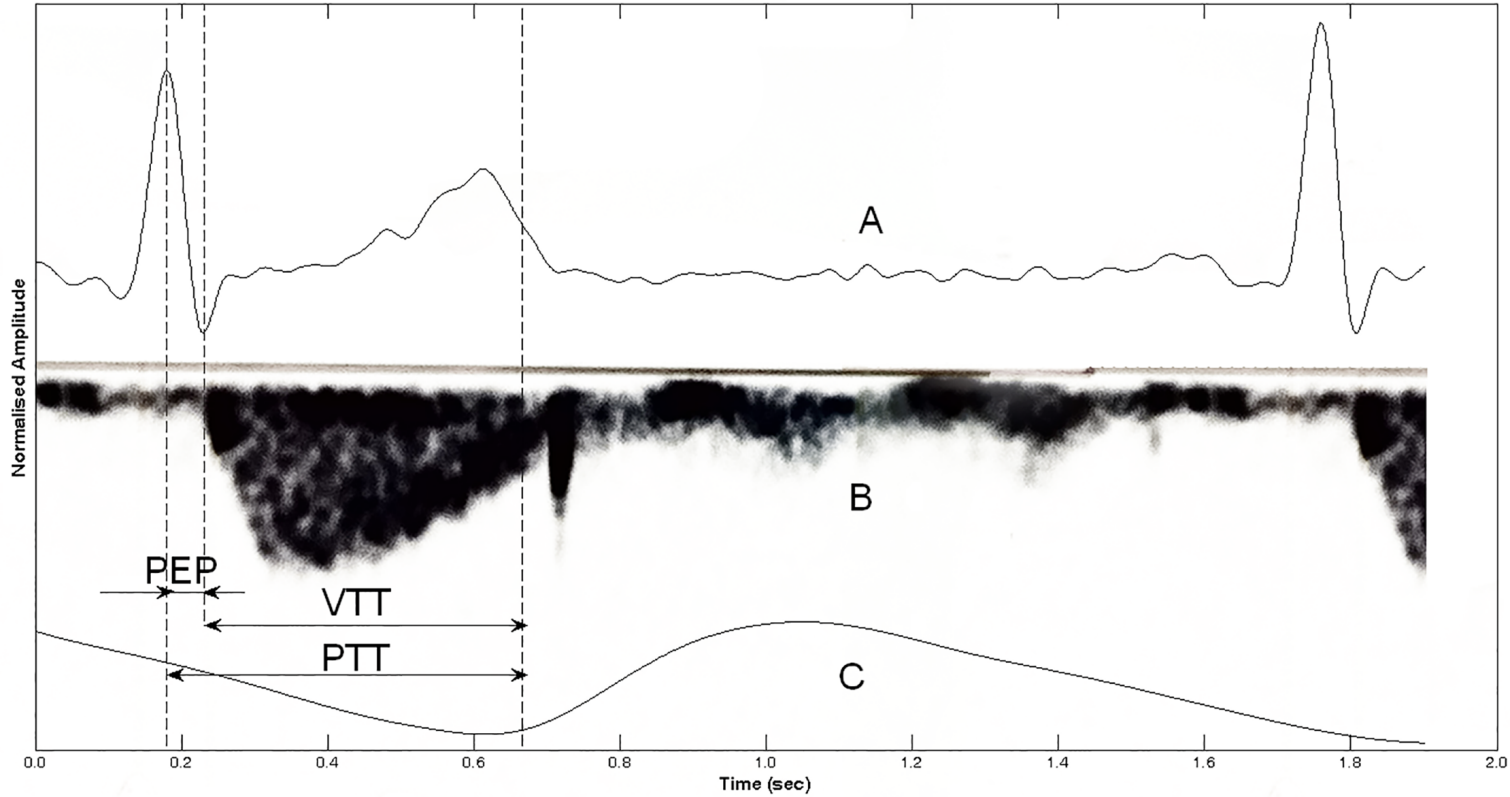
Pre-ejection period. A, electrocardiogram; B, Doppler mode echocardiography signal over the aortic valve; C, photoplethysmographic pulse wave. PEP = pre-ejection period, VTT = vascular transit time, PTT = pulse transit time.

PTT is defined as the time between the R-wave on the electrocardiogram (ECG) and the arrival of the resulting pulse wave measured with photoplethysmography (PPG). The PEP is a component of the PTT measurement (Fig 1).

The PEP represents the isovolumetric contraction time of the ventricles of the heart. It is the time between electrical activation of the ventricles (Q-wave) and opening of the aortic valve [13]. The R-wave is commonly used instead of the Q-wave [14]. Identification of R-waves is easier and more reliable than Q-waves. Moreover, Seery et al. showed that PEP can be calculated using the R-wave instead of the Q-wave [15]. PEP can be considered as a measure of left ventricular function, as it reflects changes in the contractility of myocardium, left ventricular end-diastolic volume, and aortic diastolic pressure [13, 16–18]. In patients with diabetic chronic kidney disease, fluid overload is a marker for left ventricular systolic dysfunction and is associated with the ratio of brachial PEP and brachial ejection time [19]. Physical activity, as well as respiration or a stress response, can rapidly change the PEP [20, 21]. Therefore, a change in either PEP and/or VTT can result in a change in the PTT value [22].

Cardiothoracic impedance is a measurement that is often used to acquire data for calculation of the PEP and measured PEP values around 75–134 ms [14, 23,24]. However, echocardiography is the gold standard for measuring the PEP [13]. Muehlsteff et al. investigated the role of the PEP on PTT [4]; they measured the PEP in a small group of young healthy volunteers with thorax impedance after exercise and found that PEP dominates the PTT variability after exercise. The use of different measuring tools introduces a discrepancy in literature about the magnitude of PEP and its contribution to the PTT.

The aim of this study was to estimate the variability of PEP at rest, to establish how accurate PTT is as an approximation of VTT. Age and cardiovascular risk factors (arterial hypertension, dyslipidemia, kidney failure, and diabetes mellitus) can increase arterial stiffness and, thereby, decrease VTT and possibly influence the PEP. Therefore, to take these factors into account, the PEP was measured in a control group with no medical history of cardiovascular disease aged ≤50 years and aged ≥50 years, and in a group of persons with at least one cardiovascular risk factor.

## Methods

This single-center prospective observational study examined the variability of PEP at rest in 60 volunteers divided into 3 groups (20 per group):

1. a control group of individuals without a medical history of cardiovascular disease aged under 50 years
2. a control group aged over 50 years, and
3. a group of individuals with at least one cardiovascular risk factor, which are defined in the exclusion criteria for the control groups.

The study was approved by the Medical Ethical Committee of the Erasmus University Medical Centre in Rotterdam (MEC-2011-213), and was conducted in accordance with the Declaration of Helsinki. The primary endpoints of the study were PEP, VTT and PTT.

Volunteers were eligible for inclusion when aged ≥18 years and ≤ 75 years. Exclusion criteria were arrhythmia, tremor, muscle or skeletal injuries in upper limb, hematopoietic disease, and incapacitated subjects. Further exclusion criteria for the control group (groups 1 and 2) were history of cardiovascular or peripheral vascular risk factors; these were defined as arterial hypertension, dyslipidemia, kidney failure, and diabetes mellitus. All participants were informed about the aim of the study and gave written informed consent before being enrolled. None of the eligible volunteers refused inclusion in the trial.

### Measurement protocol

All measurements were performed under standardized stable conditions in a quiet temperature- controlled room with dimmed light. First, the participant had a short interview to acquire the data such as weight, height and medication and blood pressure was measured. After that the participant was asked to lie down in a left lateral decubitus position and was connected to the measurement equipment (MP100, Biopac Systems, Inc. Goleta, USA). ECG electrodes were placed (ECG100C amplifier, Biopac) to calculate the PTT and for ECG registration during echocardiography. Four PPG sensors (TSD200 and PPG100C amplifier, Biopac) were placed on the index fingers and big toes, for measuring respectively the PTT_finger_ and PTT_toe_. Data were sampled with 2 kHz using the AcqKnowledge 3.7.3 version software (Biopac). Per volunteers, the data acquisition took place in 5 to 10 minutes.

### Echocardiography

ECG-gated echocardiography was performed at rest in the left lateral decubitus position by an experienced investigator, using a Vivid-i portable ultrasound system (GE, Solingen, Germany). Measurements were performed during 10 consecutive heartbeats, simultaneous with PTT measurements. A continuous wave Doppler registration through the aortic valve was obtained from a standard apical 5-chamber view. The horizontal sweep was set to 200 mm/s for maximum accuracy. PEP was defined as the time measured from the R-wave of the ECG to the start of the ejection phase. The data were synchronized using the matching R-waves of the simultaneously acquired second lead ECG signals from the echocardiography and PTT measurements.

### PTT calculations

Data from the AcqKnowledge software were imported into Matlab R2010a (The MathWorks, Inc., Natick, MA, USA) with the Loadacq syntax for Matlab [25]. In the ECG data, the R-waves were detected using the Rpeakdetect syntax available from the ECGtoolbox [26]. The PPG data were filtered using a fourth-order low-pass Butterworth filter with a cut-off frequency of 9 Hz. For filtering we used a zero-phase digital filtering by processing the input data, PPG, in both the forward and reverse directions. This gives a zero phase delay and is based on a method described by Oppenheim et al. [27].The signals from the PPG were digitally cut between the R-wave and the next R-wave. Thereafter the pulse waves were selected by a ‘7Step PW-filter’ to filter out the pulse waves that strongly deviated in shape for a suitable pulse wave analysis [28]. PTT was determined by calculating the time between the R-wave of the ECG and the foot of the PPG pulse wave. The foot was determined as the maximum value of the second derivative of the pulse wave [29].

### Analyses

We calculated the mean PEP of the 10 consecutive heart beats for each participant and of the total group. All data are presented as mean ± standard deviation (SD). Furthermore, to show the magnitude of the contribution of PEP and its variation in the PTT measurements in an individual, the PTT_Mean,_ PTT_SD_, PEP_Mean_ and PEP_SD_ were normalized to a percentage of the PTT_Mean_ by the following formula (see formula 1):

$$PTT_{SD_{\%}}=\frac{PTT_{SD}}{PTT_{Mean}}*100\%$$ (1)

A similar calculation was used to determine the PEP_Mean%_ and PEP_SD%._ The values of the left and right sensors were compared with the Wilcoxon signed rank test. The difference in PEP and study population characteristics between the 3 groups was tested with ANOVA. To correct for the influence of systolic blood pressure on PTT the ANCOVA was used. A *p*-value <0.05 was considered statistically significant. Statistical analyses were performed using SPSS version 24 (SPSS Inc., Chicago, USA). Figures were made using GraphPad Prism version 5.00 for Windows (GraphPad Software, San Diego California USA) and Adobe Illustrator (Creative Suits 5 CS5, Adobe Systems Incorporated, San Jose, California, USA).

## Results

The present study included 60 volunteers, divided into 3 groups of 20 participants each. As shown in Table 1, the mean age in group 1 is significantly lower than in the other groups. This is mainly caused by the age limit of 50 years in group 1. Also, the BMI was lower in the control group compared to group 2 and 3 and the mean heart rate was higher. The mean systolic blood pressure of group 3 was significantly higher than that of groups1 and 2 (*p* = 0.048). Of all the participants in group 3, 19 had risk factor arterial hypertension, 13 dyslipidemia, 2 diabetes and 1 kidney failure. The characteristics of the study population are presented in Table 1.

**Table 1:** Characteristics of the study population.

Variables	Group 1	Group 2	Group 3
Age, years	27 ± 4*	55 ± 4	63 ± 10
Sex (male), no. (%)	9 (45)	10 (50)	10 (50)
Weight, kg	69 ± 12	78 ± 12	74 ± 12
Height, m	1.77 ± 0.11	1.75 ± 0.09	1.71 ± 0.09
Body Mass Index, kg/m^2^	21.8 ± 2.5*	25.5 ± 3.6	25.3 ± 3.2
Blood pressure, mmHg			
Systolic	131 ± 13	131 ± 17	141 ± 15*
Diastolic	81 ± 10	79 ± 9	81 ± 8
MAP, mmHg	98 10	97 ± 10	101 ± 9
Heart rate, beats/min	79 ± 21*	67 ± 10	68 ± 14

n = 20 per group. Group 1 = control group <50 years, group 2 = control group >50 years, group 3 = participants with a cardiovascular risk factor. Data are presented as mean ± SD or number (%) of participants. MAP = mean arterial pressure.

* *p*<0.05

**Fig 2:**
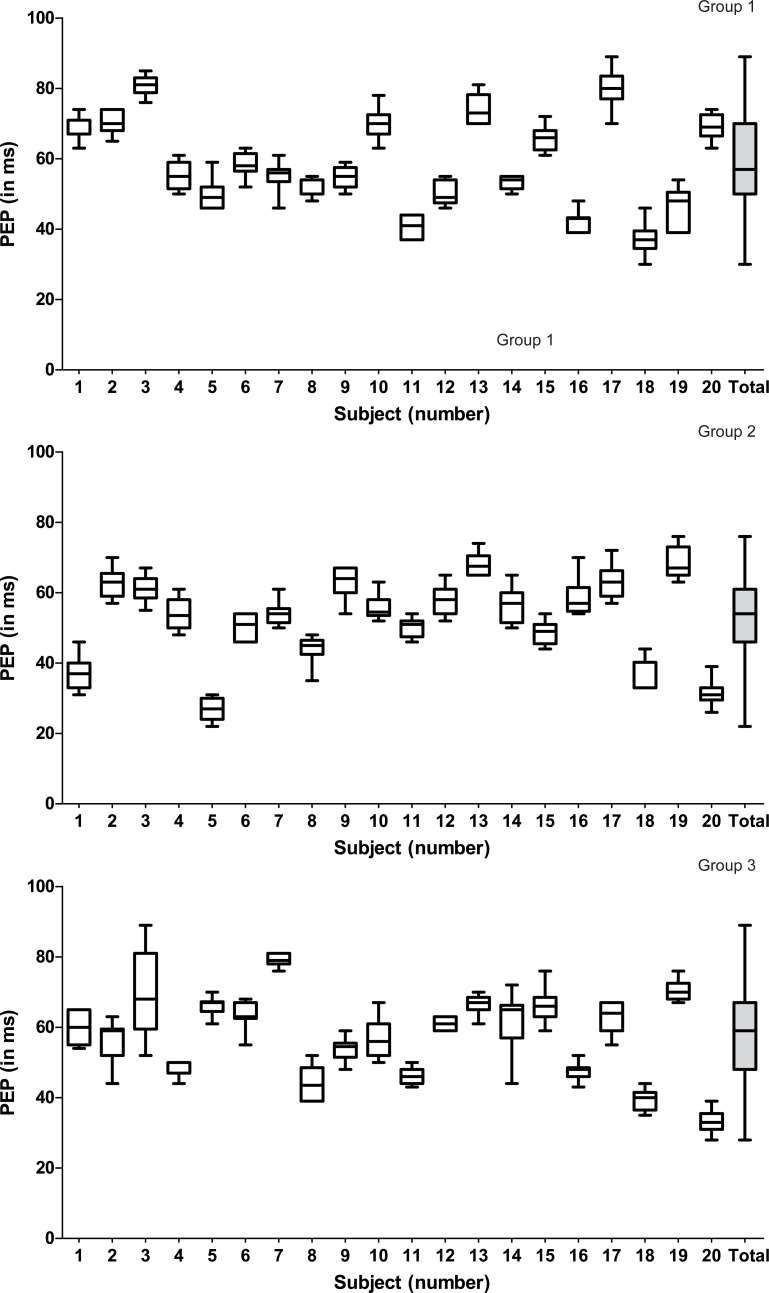
Box plots of the pre-ejection period (PEP) of 10 consecutive heartbeats of the 20 participants and of the total group. Group 1: control group aged <50 years. Group 2: control group aged >50 years. Group 3: participants with a cardiovascular risk factor. The boxes represent the median with interquartile range. The whiskers represent the minimum and maximum value.

In the total group of 60 participants, the mean PEP was 56.2 ± 12.3 ms. In groups 1, 2 and 3 the mean PEP was 58.5 ± 13.0 ms, 52.4 ± 11.9 ms and 57.6 ± 11.6 ms, respectively. The intra-individual SD of the PEP was much smaller, i.e. 2.0–5.9 ms (group 1), 2.8–5.1 ms (group 2) and 1.6–12.0 ms (group 3) (Fig 2). There were no missing data in the PEP measurement and no significant difference in the mean PEP of the 3 groups (*p* = 0.235).

**Fig 3:**
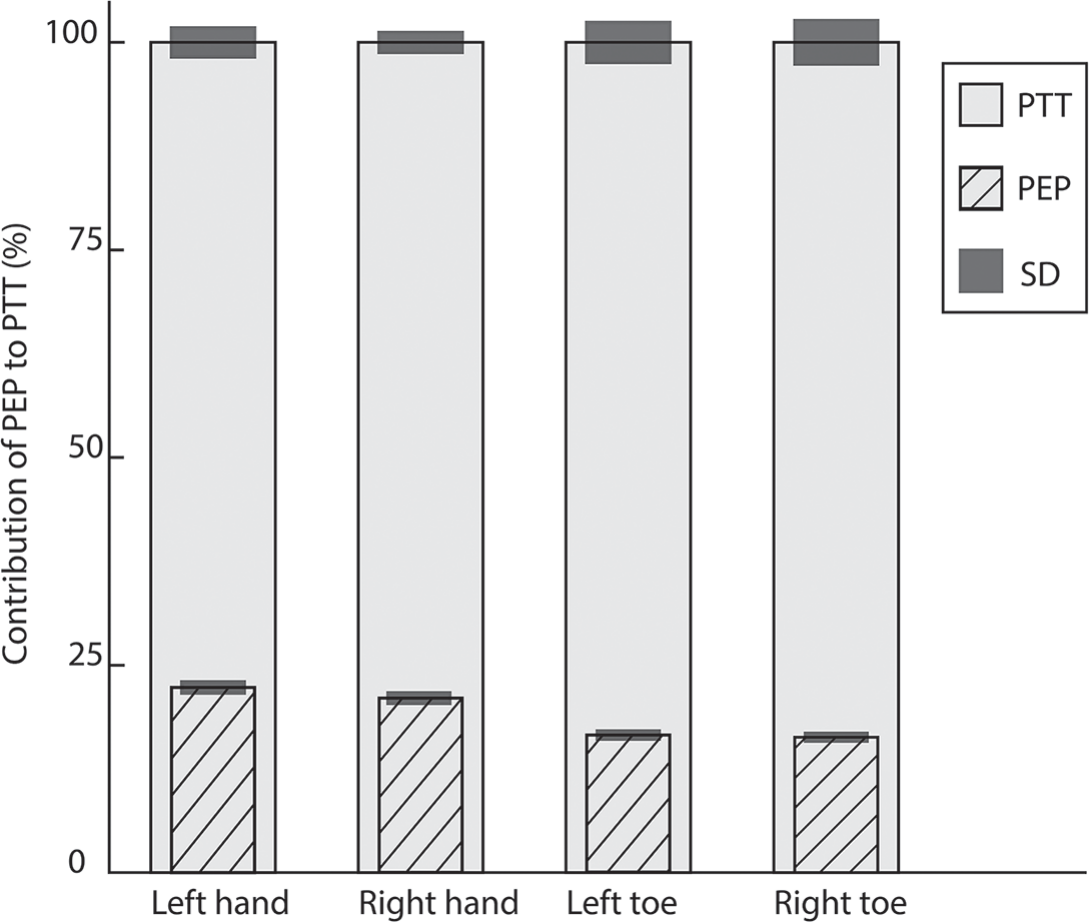
Contribution of the PEP (mean and SD) to the PTT for each sensor of group 1 (control group aged <50 years). All values are normalized to a percentage (%) of the mean PTT (which is 100%) of the specific sensor. PTT_Mean%_ of left hand (n = 19) 100%±3.1%, PTT_Mean%_ of right hand (n = 20): 100%±2.4% (*p* = 0.004). Contribution of PEP_Mean%_ for PTT of left versus right hand 22.2%±1.3% versus 21.0%±1.3%. PTT_Mean%_ of left big toe (n = 18) 100%±4.7%, PTT_Mean%_ right big toe (n = 17) 100%±4.8%. Contribution of PEP_Mean%_ for left big toe versus right big toe 16.5%±1.0% versus 16.3%±1.0%.

**Fig 4:**
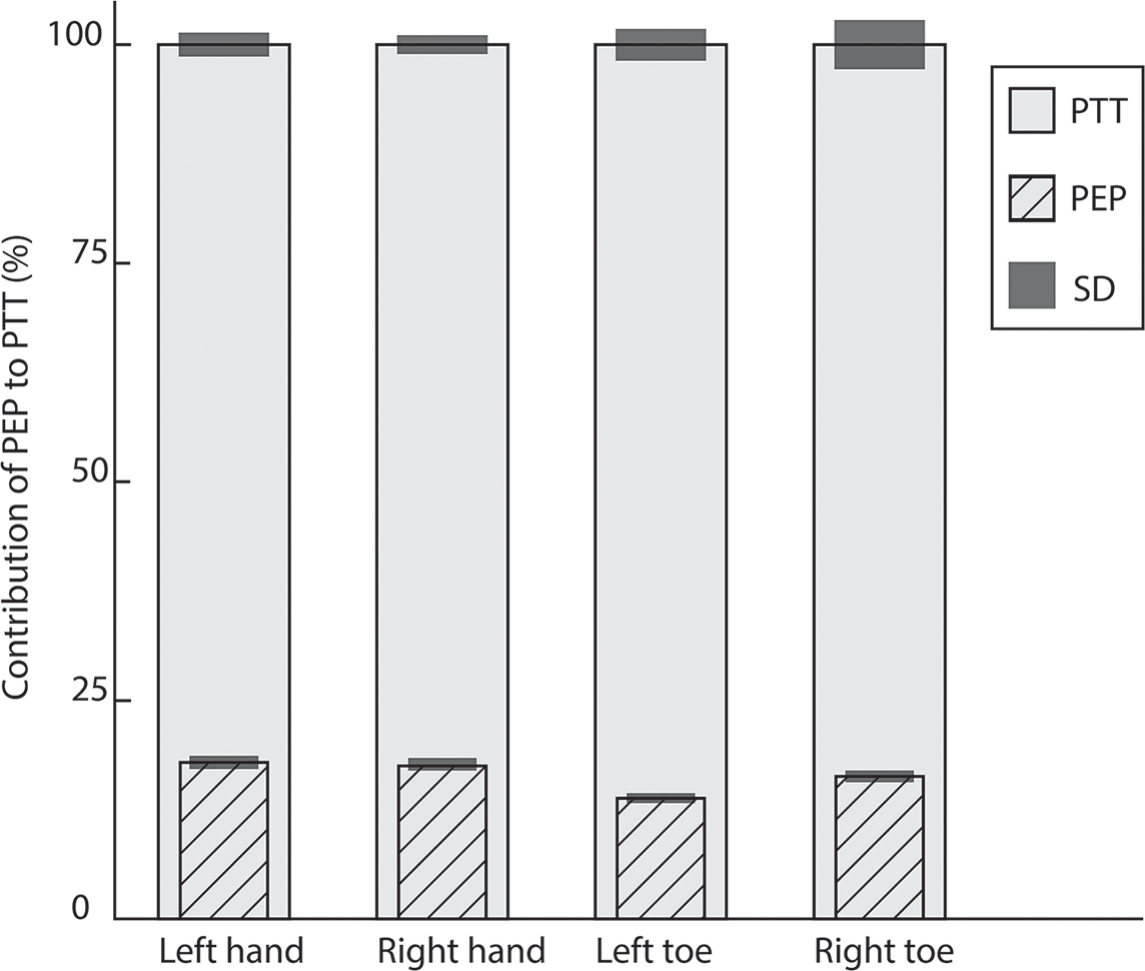
Contribution of the PEP (mean and SD) to the PTT for each sensor of group 2 (control group aged >50 years). All values are normalized to a percentage (%) of the mean PTT (which is 100%) of the specific sensor. PTT_Mean%_ of left hand (n = 19) 100%±3.5%, PTT of right hand (n = 20) 100%±2.6%. Contribution of PEP_Mean%_ for PTT of left versus right hand PEP 18.7%±1.4% versus PEP 19.4%±1.4%. PTT_Mean%_ of left big toe (n = 18) 100%±5.9%, PTT_Mean%_ right big toe (n = 17) PTT 100%±6.3%. Contribution of PEP_Mean%_ for left big toe versus right big toe 4.8%±1.1% versus 5.1%±1.1%.

**Fig 5:**
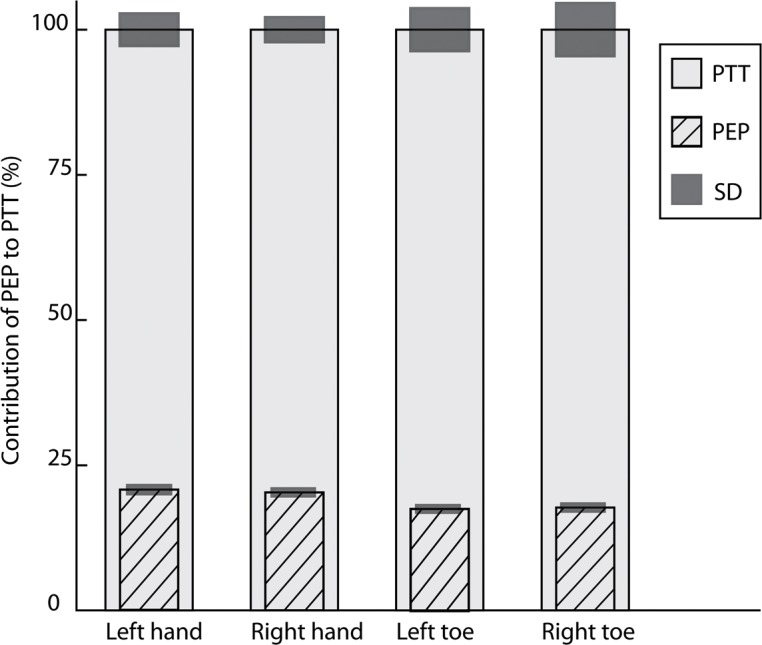
Columns represent the contribution of the PEP (mean and SD) to the PTT for each sensor of group 3 (participants with a cardiovascular risk factor). All values are normalized to a percentage (%) of the mean PTT (which is 100%) of the specific sensor. PTT_Mean%_ of left hand (n = 19) 100%±5.5%, PTT of right hand (n = 20) 100%±4.2%. Contribution of PEP_Mean%_ for PTT of left versus right hand 20.7%±1.5% versus 20.3%±1.4%. PTT_Mean%_ of left big toe (n = 15) 100%±7.3%, PTT_Mean%_ right big toe (n = 14) 100%±9.1%. Contribution of PEP_Mean%_ for left big toe versus right big toe 17.6%±1.3% versus 17.7%±1.4%.

For all the PPG measurements of the 3 groups together, 18 sensors showed too many artifacts, modulated waves, for reliable PTT calculation; these measurements were excluded from the analysis, which represents 7.5% of the data (for specification see Figs 3, 4 and 5). Pulse waves were only excluded from analyses when they did not pass the ‘7Step PW-filter’.

In the normalized data of group 1, the PEP_Mean%_ contribution to the PTT_Mean%_ of the left hand was significantly different compared to the right hand (*p* = 0.004) (Tables 2 and 3) (Fig 3). This result is still significant after applying the Bonferroni correction for multiple testing, *p*<0.008. In groups 2 and 3 there was no significant difference between the left and right measurements (Figs 4 and 5). Between the groups was no significant difference in the contribution of PEP_Mean%_ to the PTT_Mean%,_ neither after correction for SBP.

**Table 2:** Data on normalized PTT and PEP variability.

	Normalized PTT in % (PTT mean in ms)	SD PTT in % of PTT (PTT SD in ms)	% of PEP with respect to PTT	% of SD PEP with respect to PTT
**1**	100 (305)	1.6 (5)	22.4	1.1
**2**	100 (272)	2.6 (7)	25.8	1.1
**3**	100 (285)	2.5 (7)	28.3	0.9
**4**	100 (299)	1.7 (5)	18.5	1.3
**5**	100 (309)	1.9 (6)	16.1	1.3
**6**	100 (268)	1.1 (3)	21.8	1.3
**7**	100 (266)	1.9 (5)	20.7	1.5
**8**	100 (286)	1.0 (3)	17.9	0.8
**9**	100 (230)	1.7 (4)	23.8	1.3
**10**	100 (270)	1.9 (5)	25.9	1.5
**11**	100 (272)	1.1 (3)	14.9	1.2
**12**	100 (274)	0.7 (2)	18.3	1.2
**13**	100 (247)	4.5 (11)	29.9	1.7
**14**	100 (271)	3.3 (9)	19.6	0.7
**15**	100 (292)	1.0 (3)	22.5	1.2
**16**	100 (240)	5.8 (14)	17.5	1.3
**17**	100 (330)	2.7 (9)	24.2	1.7
**18**	100 (294)	2.0 (6)	12.7	1.5
**19**	100 (276)	5.4 (15)	16.7	2.1
**20**	100 (315)	3.8 (12)	22	1.2
**Mean group**	100	2.4	21.0	1.3

Values are normalized as percentage (%) of the PTT sensor of the index finger of the right hand group 1 (control group <50 years). The PTT mean ± SD (in ms) of 10 consecutive heartbeats are given for each person. For calculation of the normalized data see Formula 1. In group 1 the normalized PTT showed a significant difference between the left and right hand, respectively 100%±3.1% versus 100%±2.4% (*p* = 0.004).

**Table 3:** Data on normalized PTT and PEP variability per group.

	Normalized PTT in % (PTT mean in ms)	SD PTT in % of PTT (PTT SD in ms)	% of PEP with respect to PTT	% of SD PEP with respect to PTT
**Group 1**				
Left hand	100 (267)*	3.1 (8)	22.2	1.3
Right hand	100 (280)*	2.4 (7)	21.0	1.3
Left foot	100 (369)	4.7 (17)	16.5	1.0
Right foot	100 (375)	4.8 (18)	16.3	1.0
**Group 2**				
Left hand	100 (276)	3.5 (12)	18.7	1.4
Right hand	100 (275)	2.6 (7)	19.4	1.4
Left foot	100 (359)	5.9 (21)	14.8	1.1
Right foot	100 (350)	6.3 (20)	15.1	1.1
**Group 3**				
Left hand	100 (280)	5.5 (16)	20.7	1.5
Right hand	100 (284)	4.2 (12)	20.3	1.4
Left foot	100 (339)	7.3 (22)	17.6	1.3
Right foot	100 (336)	9.1 (25)	17.7	1.4

Mean values per group, n = 20 per group. Group 1 = control group participants aged <50 years, group 2 = control group participants aged >50 years, group 3 = participants with a cardiovascular risk factor. Values are normalized as percentage (%) of the PTT sensor.

* *p*<0.05

## Discussion

This study aimed to determine the variability of PEP at rest and its contribution to PTT in order to assess the accuracy of measuring VTT with PTT. Since it is difficult to measure PEP with echocardiography during exercise, we measured the PEP at rest only. The absolute value of the variability and SD of the PEP between individuals at rest was large when compared with the variability in one participant. However, in the normalized data, the SD of the PEP is only approximately 1.0–1.5% of the PTT. In an earlier study, we found an increase of the PTT of 17 ms after a successful axillary block from 259 ms to 276 ms [30]. This change is larger than can be expected to be caused by a variation of PEP alone.

This study has some limitations. The participants of this study did not refrain of caffeine, neither for physical activity for 48 hours. This might have an effect on the measurements. Nonetheless, in the study of Kohler et al caffeine had no significant effect on the PEP compared to the baseline measurements [31]. However, after exercise, the hemodynamic stress response is reduced and causes an significant increase in PEP [32]. Moreover, we did not control for the estrogenic phase of the participants. In this study 50% of the participants is female and the female participants in group 2 and 3 are above 50 years old and most likely postmenopausal. Farinatti et al showed in their study that the PEP was similar across age groups in men and women [33].

Furthermore, the PTT was measured bilaterally and the difference between the blocked arm and the contralateral arm was calculated. Other studies have shown that PTT difference is a method to exclude the effect of PEP and can be used to monitor arterial distensibility or pulse wave velocity changes [34]. Therefore, the increase in PTT in the present study is most probably a result of the vascular component of the PTT, the VTT.

Furthermore, in group 1, the contribution of the PEP to the PTT_finger_ was 22.3% and 21.0% for the left and right hand, respectively; this was a significant difference between the left and right hand. This can be explained by the difference in PTT of the left and right hand. In our earlier study, we found no difference between the PTT of the left and right hand in a person at rest in supine position [30]. For an optimal apical 5-chamber view with echocardiography, a left lateral decubitus position was required with the left hand positioned above their head. PTT is related to the position of the arms [35, 36]. Moreover, this position can introduce a different curvature of the artery to the arm. Therefore, the left lateral decubitus position is a possible explanation for this difference in PTT and, subsequently, the contribution to PTT. However, there was no significant difference between the left and right hand in groups 2 and 3.

The contribution of the PEP to the PTT_toe_ was smaller compared to the PTT_finger_. This difference can be explained by the length of the arterial pathway to the toes and a subsequently longer VTT (and PTT).

In this study, the mean PEP of all 3 groups was 56.2 ± 12.3 ms; this differs from the values found in other studies. The main reason for this is the diverse acquisition techniques used and/or an estimation of PEP made by measuring the left ventricular ejection time [17]. Johansson et al. investigated the use of PTT for respiration rate monitoring; PTT varies with respiration. The authors investigated whether measuring the individual components of the PTT (PEP and VTT) could improve their respiration detection [21]. The PEP was measured with phonocardiography, by using the sound of the closing valves. For technical reasons, they used the first heart sound (S1) which represents the closing of the mitral valve, instead of measuring the opening of the aortic valve. However, after the mitral valve closes (S1) and the isovolumetric contraction starts, the ventricular pressure will rise. When the ventricular pressure is high enough, the aortic valve opens and the ejection of blood to the aorta starts. Therefore, using phonography will introduce an inaccuracy in the measurement. They found a mean PEP at rest of 30.1 ms with a SD of 8.0 ms. Furthermore, the PEP and VTT varied synchronously during respiration; focusing on these components did not improve their respiration detection.

In most of the studies, the PEP was measured with cardiothoracic impedance, which measures the change in blood volume in the thorax, which is primarily caused by the blood flow in the aorta. The advantage of cardiothoracic impedance is that it does not require specially trained personnel and can be measured in every body position and also during movements. It is an indirect measurement and is less accurate than a direct measurement and gold standard echocardiography [37], which was used in the present study. Furthermore, these measurements were performed in three different groups. Age and cardiovascular risk factors such as hypertension and atherosclerosis are important factors for PTT. They influence the stiffness of arteries and can change the contribution of PEP to the PTT. However, our measurements showed no significant difference in the PEP between the groups; moreover, the PEP_SD%_ was very small (between 1.0 and 1.5).

## Conclusion

In conclusion, the contribution of PEP to the PTT measured at the finger tips at rest is approximately 20%. Therefore, the VTT is represented by the remaining 80% of the PTT. Since the PEP variability within an individual at rest is small (PEP_SD%_ 1.0–1.5% of the PTT value), its contribution to a change in PTT is also small. Therefore, PTT is an attractive method to non-invasively monitor arterial stiffness at rest. Furthermore, unlike methods such as Doppler ultrasound, it does not need specially trained personnel. ECG and PPG (pulse oximeter) are already available in hospitals and software implementation in the monitors is needed to enable PTT calculation. Therefore, PTT at rest is an easy, non-invasive, low-priced measurement, and accurate approximation of VTT for monitoring arterial stiffness.

## Supporting information

S1 Database PEP data supporting study findings. (SAV)
